# Long-Term Physical Exercise and Mindfulness Practice in an Aging Population

**DOI:** 10.3389/fpsyg.2020.00358

**Published:** 2020-04-02

**Authors:** Yi-Yuan Tang, Yaxin Fan, Qilin Lu, Li-Hai Tan, Rongxiang Tang, Robert M. Kaplan, Marco C. Pinho, Binu P. Thomas, Kewei Chen, Karl J. Friston, Eric M. Reiman

**Affiliations:** ^1^Department of Psychological Sciences, Texas Tech University, Lubbock, TX, United States; ^2^Institute of Neuroinformatics and Laboratory for Body and Mind, Dalian University of Technology, Dalian, China; ^3^Center for Brain Disorders and Cognitive Science, Shenzhen University, Shenzhen, China; ^4^Department of Psychological and Brain Sciences, Washington University in St. Louis, St. Louis, MO, United States; ^5^Clinical Excellence Research Center, Department of Medicine, Stanford University, Stanford, CA, United States; ^6^Department of Radiology, UT Southwestern Medical Center, Dallas, TX, United States; ^7^Advanced Imaging Research Center, UT Southwestern Medical Center, Dallas, TX, United States; ^8^Banner Alzheimer’s Institute, Phoenix, AZ, United States; ^9^Wellcome Centre for Human Neuroimaging, Institute of Neurology, University College London, London, United Kingdom

**Keywords:** physical exercise (PE), mindfulness interventions, integrative body-mind training (IBMT), quality of life, secretory Immunoglobulin A (sIgA), cortisol, heart rate variability, skin conductance response

## Abstract

Previous studies have shown that physical exercise and mindfulness meditation can both lead to improvement in physical and mental health. However, it is unclear whether these two forms of training share the same underlying mechanisms. We compared two groups of older adults with 10 years of mindfulness meditation (integrative body-mind training, IBMT) or physical exercise (PE) experience to demonstrate their effects on brain, physiology and behavior. Healthy older adults were randomly selected from a large community health project and the groups were compared on measures of quality of life, autonomic activity (heart rate, heart rate variability, skin conductance response, respiratory amplitude/rate), immune function (secretory Immunoglobulin A, sIgA), stress hormone (cortisol) and brain imaging (resting state functional connectivity, structural differences). In comparison with PE, we found significantly higher ratings for the IBMT group on dimensions of life quality. Parasympathetic activity indexed by skin conductance response and high-frequency heart rate variability also showed more favorable outcomes in the IBMT group. However, the PE group showed lower basal heart rate and greater chest respiratory amplitude. Basal sIgA level was significantly higher and cortisol concentration was lower in the IBMT group. Lastly, the IBMT group had stronger brain connectivity between the dorsal anterior cingulate cortex (dACC) and the striatum at resting state, as well as greater volume of gray matter in the striatum. Our results indicate that mindfulness meditation and physical exercise function in part by different mechanisms, with PE increasing physical fitness and IBMT inducing plasticity in the central nervous systems. These findings suggest combining physical and mental training may achieve better health and quality of life results for an aging population.

## Introduction

Aging is associated with decline in processing speed, memory, motor control and mental flexibility, which greatly impacts individual well-being and health. Consequently, there has been an increasing interest in interventions that could (a) maintain and enhance physical and mental vitality and (b) reduce the risk of age-associated physical and mental disorders in aging populations (Mahncke et al., 2006; Hillman et al., 2008). Previous studies have shown that both physical exercise and forms of mindfulness meditation can influence aspects of attention and self-regulation (Okazaki et al., 2005; Tang et al., 2007, 2009, 2010, 2015a; Hillman et al., 2008; Ludwig and Kabat-Zinn, 2008; Wen et al., 2011). However, physical exercise (PE) and mindfulness meditation are vastly different from each other, as each intervention has unique training components and may work through different mechanisms to exert an impact on behavior, physiology and brain.

As one of the most widely adopted interventions, PE requires the engagement and control of body movement and coordination with the outside environment. Months to years of PE have been shown to improve cardiovascular function, physical health and cognitive performance, and are associated with larger gray matter volumes in certain brain regions such as the hippocampus (Okazaki et al., 2005; Hillman et al., 2008; Wen et al., 2011). In contrast, mindfulness meditation involves less physical activity compared to PE, but focuses more on training attention, self-awareness, and emotion regulation through various mental techniques and strategies (Tang et al., 2015a). Studies have shown that 5 days of mindfulness meditation using integrative body-mind training (IBMT) improves attention and reduces stress reactivity through changing the interaction between the anterior cingulate cortex (ACC) and the parasympathetic branch of the autonomic nervous system (Tang et al., 2007, 2009). Furthermore, it was found that about 10 h of IBMT over one-month period increases fractional anisotropy (FA), an index indicating the integrity and efficiency of white matter in the anterior corona radiata, an important white-matter tract connecting the ACC to other structures (Tang et al., 2010). In addition, several long-term meditation studies have shown increases in ACC activation and gray matter volume and other regions, such as the striatum and insula (Cahn and Polich, 2006; Ludwig and Kabat-Zinn, 2008; Chiesa and Serretti, 2010; Tang et al., 2015a; Tang, 2017).

Given that both mindfulness meditation and PE have produced documented brain changes, we first sought to investigate how neuroplasticity in these long-term trainings would differ from each other by comparing these two forms of training in older adults. In particular, we sought to determine how and why each might contribute to more successful aging. To accomplish these goals, our design stresses comparative effectiveness and comparative mechanisms of training rather than the more usual tests of whether a given training method is effective. While many comparative effectiveness studies have been performed, our study is unique because it involves an aging population with 10 years of either mindfulness meditation or physical exercise (PE) experience.

Based on our prior studies showing improvement in self-regulation and increases in ACC and striatum activity after short-term mindfulness training (Tang et al., 2007, 2009, 2010, 2012, 2015a), our first hypothesis was that there would be stronger resting state connectivity between ACC-Striatum and greater gray matter in striatum following IBMT than following PE. For physiological indices, we hypothesized that both long-term mindfulness meditation and PE should induce changes in immunology and stress hormone based on prior literature showing such physiological benefits following short-term training using these two interventions (Tang et al., 2007; Hillman et al., 2008). In our previous studies, we found that 5 days of IBMT improves secretory Immunoglobulin A (sIgA) level following additional 20 min training after stress (Tang et al., 2007), and the baseline sIgA level is greater after 4-weeks of IBMT compare to relaxation training (Fan et al., 2010, 2013). While PE may also improve the immune function, our second hypothesis was that the IBMT group would show greater or at least equal sIgA level when compare with the PE group.

Physiological indexes are important biomarkers for aging (Pumprla et al., 2002; Okazaki et al., 2005; Borresen and Lambert, 2008). Heart rate, heart rate variability (HRV), skin conductance response (SCR), respiratory amplitude and rate were used to assess autonomic nervous system (ANS) activity (Tang et al., 2009). Because IBMT changes the state of the body and mind through the interaction of central and autonomic nervous systems, and PE strengthens cardiovascular activity and physical fitness, our third hypothesis was that PE would have lower resting heart rate than IBMT, whereas IBMT would show better ANS regulation indexed by lower SCR, and greater high frequency HRV in comparison to PE.

Finally, to compare quality of life outcomes following 10 years of PE or IBMT, we used the widely validated World Health Organization Quality of Life Survey (WHOQOL-100) to evaluate physical and psychological domains (Power et al., 1999). Because IBMT improved mood and showed greater activation in reward associated brain structures (Tang et al., 2007, 2009, 2010, 2015a), we hypothesized that higher quality of life would be associated with IBMT.

## Materials and Methods

### Study Population

Two groups of (IBMT:PE = 32:29; 17 males) healthy Chinese older adults (mean age = 64.25 years old, SD = 12.54) were randomly chosen from a longitudinal National Health Project in the local communities based on their willingness to participate in our behavior, physiology and brain imaging study. All participants were living independently in their own home with matched living environment and social-economic status and were free of psychiatric disability and dementia assessed by local clinicians using semi-structured clinical interviews. Participants were not habitual smokers or drinkers, did not take any psychotropic drugs, anti-depressants or cholinesterase inhibitors within the last 12 months. Participants also received an annual health/medical check in the community hospitals. The two groups were matched by level of education, age and sex, see Table 1. No sedentary control group was assigned, because (1) the majority of Chinese old adults exercise to some degree after retirement and (2) such a waitlist group would be unlikely to maintain good health over a 10-year period. All subjects provided written informed consent in accordance with the Declaration of Helsinki. The study protocol was approved by the Institutional Review Board of the Dalian University of Technology.

**TABLE 1:** Demographic information for participants.

Demographic factor	IBMT		PE		*P* value (two-tailed)
	Mean	*SD*	Mean	*SD*	
Age	64.38	13.95	64.13	11.19	0.94
Education	13.89	2.79	12.71	3.74	0.19

### Interventions

China belongs to a collectivist culture and old adults after retirement often get together for shared daily activities in local communities (Goodwin, 1999; Nisbett, 2004; Markus and Conner, 2014). The participants were trained in small groups in local communities. Each group practiced PE or IBMT daily for an average of 1 h, usually 6–7 days per week, for a 10-year period. Although some were absent from some training sessions, the average training duration was about 3120 h over 10 years in total. The IBMT or PE instructor in the same community supervised the practice sessions. The instructors also met and had biweekly conversations with participants to monitor and motivate the two groups and gave appropriate feedback. We found Chinese collectivist culture helped facilitate group practice in old adults, and the first 3-month of practice was crucial for habit formation in either IBMT or PE group. After this period, participants per group seemed to maintain independent practices regularly. Three participants in IBMT or PE group dropped off the study and in total 55 participants (29 in IBMT group) completed.

#### Integrative Body-Mind Training

Mindfulness Meditation involves paying attention to the present and increasing awareness of one’s thoughts, emotion, and actions without judging oneself. One form of mindfulness meditation, IBMT involves body relaxation, mental imagery and mindfulness training which are the common components in other mindfulness programs (Kabat-Zinn, 2013). All sessions started and ended with approximately 10 min of gentle and slow posture practice for the purpose of warming up and returning to normal states. Cooperation between body and mind is emphasized in facilitating and achieving a meditative state. The instructor helped participants find an appropriate and balanced body posture to achieve deeper levels of relaxed body and experience a quietness and mindfulness state. When the mind wanders, participants accepted and were open to these experiences without judgment. IBMT uses minimal effort to control thoughts, but instead establishes a state of restful alertness that allows a high degree of awareness of body, breathing, and external instructions. It stresses a balanced state of relaxation while focusing attention on the present moment (Tang et al., 2007, 2015a; Tang, 2017).

#### Physical Exercise

Training mainly involved aerobic walking. All walking sessions started and ended with approximately 10 min of stretching for the purpose of warming up and cooling down. The participants were encouraged to walk in their target heart rate zone following light to moderate intensity exercise, which was calculated based on the resting and maximum heart rates achieved during the baseline maximal graded exercise test (Strath et al., 2000). For example, if the participant was 60 years old, the target heart rate zone was from 80 to 112 beats per minute during moderate intensity exercise.

### Outcome Measures

#### Brain Imaging

Brain imaging experiments were performed on a SIEMENS TrioTim 3 Tesla scanner (Siemens Medical System, Erlangen, Germany) at the MRI Center for Brain Research. The resting state session consisted of 180 contiguous echo planar imaging (EPI) volumes with TR/TE = 2000/30 ms; flip angle = 90^0^, matrix = 64 × 64, axial, slice thickness = 4 mm. A high-resolution T1-weighted anatomical image was also obtained after the resting state.

Data preprocessing of the resting state image, including slice timing, realignment, coregistration, normalized and spatially smoothed steps (6-mm full width at half maximum Gaussian blur) using SPM 8^1^. Subsequent processing includes temporal band-pass filtering (0.009 < f < 0.08) typically performed by resting state fMRI data analysis (Fox et al., 2005).

To quantify (changes in) functional connectivity, subject specific (partial) Pearson’s correlation coefficient maps were computed based on the temporal series from a spherical region of interest (ROI) (radius = 6 mm) centered at the coordinates (*x* = 8, *y* = 7, *z* = 38) within the dACC brain area. These correlations were adjusted for confounds using (six) parameters obtained by rigid body correction of head motion, global trend, and signal from non-gray matter (Fox et al., 2005; Castellanos et al., 2008). Following Z-transformation of the correlation coefficients, we identified regions showing a significant functional connectivity with the dACC across both groups. Within these regions, we tested for the orthogonal effect of group differences using a small volume correction (SVC) for multiple comparisons.

To quantify (changes) in functional anatomy, voxel-based morphometry (VBM) was applied to high-resolution (1 × 1 × 1 mm) T1-weighted whole-brain images, collected from every subject with TR/TE/TI = 2530/3.37/1100 ms, NEX = 1, flip angle = 7°, matrix size = 512 × 512, Sagittal, slice thickness = 1.33 mm. A unified segmentation/normalization framework in the SPM 8 and VBM toolbox^2^ were used for VBM analysis (Ashburner and Friston, 2000).

We examined regional gray matter differences between groups using a general linear model with age and gender as covariates. The voxel-wise threshold for activation was set at *P*_FWE_ < 0.05, corrected for the number of resolution elements in each of the regions of interest (ROI) by using the SPM small volume correction (SVC) procedure together with brain masks defined by the automated anatomical labeling toolbox (AAL)^3^. The brain masks defined the brain regions over each of which the SVC was performed. These brain regions included the insula, putamen and caudate, hippocampus, frontal, temporal and parietal cortex based on the physical exercise and mindfulness meditation literatures (Greenwood, 2007; Hillman et al., 2008; Tang et al., 2009, 2010, 2015a). Twenty-six subjects (13 in each group) met all criteria for participating in the analysis, including no metallic implants and usable data after motion correction.

#### Physiology

The physiological data were recorded and analyzed in 8 channels of the Procomp Infiniti System from Thought Technology (Tang et al., 2009). During the fitness sessions, participants’ heart rate was monitored using a Polar E200 heart rate monitor. We first recorded the 5-min baseline (eyes open, labeled as Baseline 1 in Figures 3A,B) heart rate, SCR, abdomen and chest respiratory amplitude for each subject in the two groups. To attain steady physiological signals of SCR and HRV allowing measurement of habitual ANS activity/regulation, we then recorded two 10-min periods of rest (eyes closed) (labeled as 1 and 2 in Figures 3A,B), and 5-min post baseline (eyes open, labeled as Baseline 2 in Figures 3A,B).

Power spectral analysis of HRV was performed with a fast Fourier transform and Biograph software, and spectral components were identified and assigned, on the basis of their frequency: high frequency (HF; 0.16–0.45 Hz), low frequency (0.04–0.15 Hz), and very low frequency (VLF; 0-0.03 Hz). These components were obtained in absolute values of power (ms^2^). HF components are reported in normalized units (nuHF), representing the relative value of the power of each component in proportion to the total power minus the VLF component (Okazaki et al., 2005; Tang et al., 2009). Forty participants (20 in each group) participated throughout the tests, 40 had usable heart rate and SCR data, 36 had usable chest respiratory data (18 in each group) and 35 had useable HRV data (18 in IBMT group) after movement artifacts were eliminated.

#### Salivary sIgA and Cortisol

Salivary sIgA and cortisol levels were assessed at three stages (rest, stress, and additional 20-min practice, labeled as before stress, after stress and additional training respectively in Figure 4). To control for variations of sIgA and cortisol levels over the circadian rhythm, saliva sample collection was performed from 2:00 pm to 6:00 pm. About 1-mL saliva samples were collected by one-off injectors and were encased in test tubes in succession, with the tubes placed into a refrigerator under-208C and then thawed 24 h later for analysis. The concentration of sIgA and cortisol was analyzed by radioimmunoassay at the Dalian Medical University. Intra- and inter-assay coefficients of variation were below 10%. To reduce error variance due to imprecision of the intra-assay, all samples from each subject were analyzed in the same run (Tang et al., 2009).

#### World Health Organization Quality of Life Survey (WHOQOL-100)

This scale was developed in 15 international centers using focus groups, pilot tests, and field tests. The final 100 items were grouped into one scale score assessing overall quality of life and general health perceptions, and 24 quality of life facets, grouped into six larger domains: Physical, Psychological, Independence, Social Relationships, Environment, and Spirituality. The international and multicultural aspects of this scale make it a very useful instrument. It has been used extensively in a variety of settings around the world, demonstrating excellent reliability and validity (Power et al., 1999).

## Results

**FIGURE 1:**
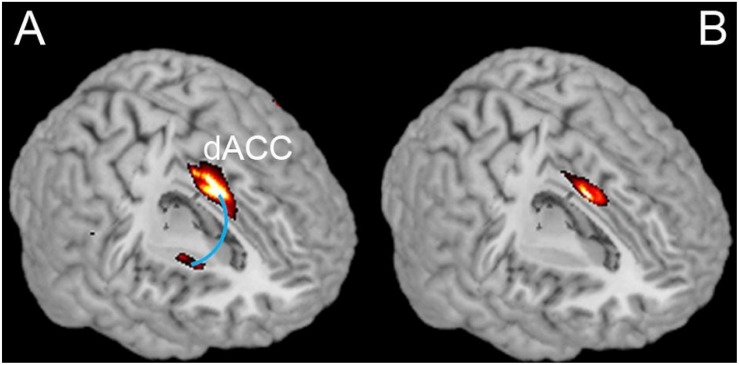
Comparison of resting state dACC-Striatum connectivity and differences between IBMT and PE. **(A)** Shows the functional connectivity between dACC and Striatum detected in both groups combined. **(B)** Shows the differences between groups in terms of a significantly greater functional connectivity in the IBMT group relative to the PE group (*P*_FWE_ < 0.05, small volume corrected).

**FIGURE 2:**
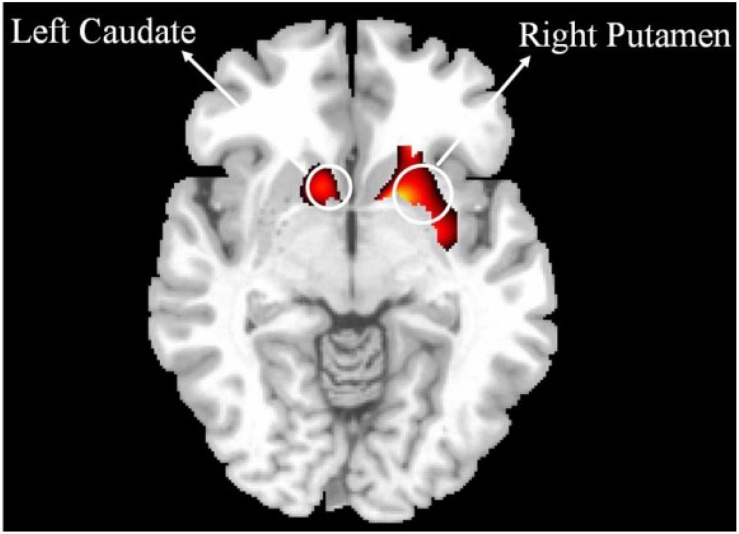
Comparison of VBM gray matter between IBMT and PE. The voxelwise threshold for activation was set at *P*_FWE_ < 0.05, corrected for the number of resolution elements in each of the regions of interest (ROI) by using the SPM small volume correction (SVC) procedure together with brain masks defined by the automated anatomical labeling toolbox (AAL) (http://www.fil. ion.ucl.ac.uk/spm/ext). The brain masks defined the brain regions over each of which the SVC was performed. These brain regions included insula, putamen, caudate, hippocampus, frontal, temporal and parietal cortex. For better illustration, a liberal threshold (*P* < 0.05, uncorrected) was used.

**FIGURE 3:**
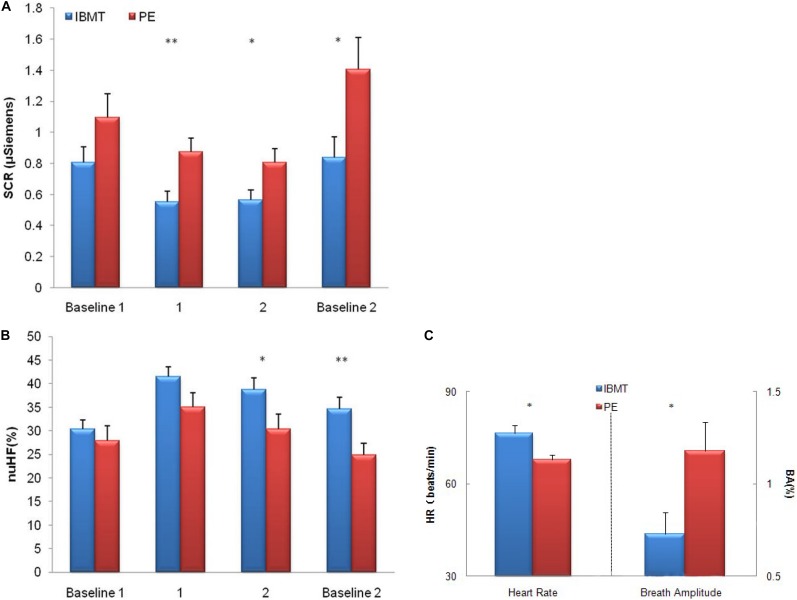
Comparison of physiological indexes between IBMT and PE. **(A)** Skin conductance response (SCR) **(B)** High-frequency HRV **(C)** Heart rate and chest respiration amplitude in the IBMT and PE groups. For SCR, the lower score shows more parasympathetic activity. For high-frequency HRV, the higher score shows more parasympathetic activity. **(A,B)** The horizontal axis indicates the four test sessions: resting with eyes open (baseline 1), two periods of resting with eyes closed (labeled as 1, 2), and resting with eyes open (baseline 2). The vertical axis indicates SCR change and percentage of change in normalized units of high-frequency (nuHF) HRV, respectively. **(C)** The vertical axis indicates resting heart rate (HR) change and percentage of change of chest breath amplitude (BA), respectively. Error bars represent standard errors. ^∗^*P* < 0.05, ^∗∗^*P* < 0.01.

**FIGURE 4:**
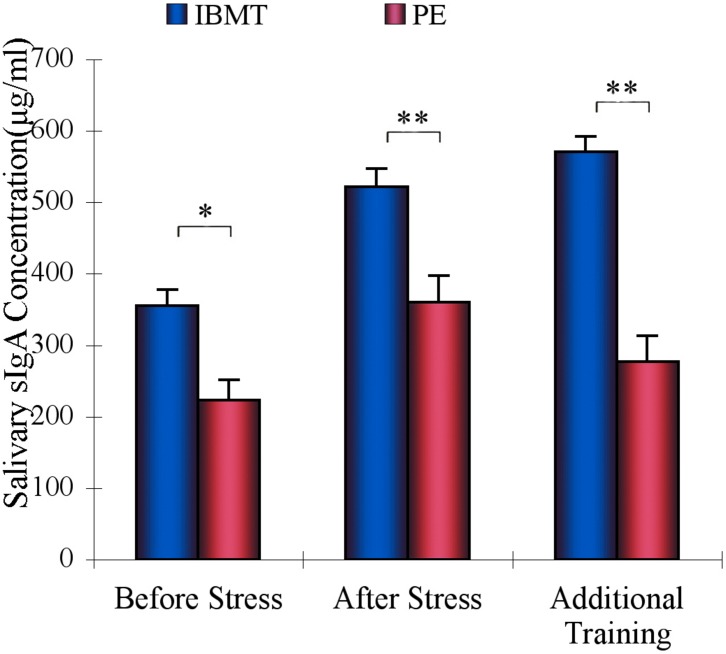
Comparison of sIgA between IBMT and PE. IBMT (blue dots) and PE (red dots) groups. The IBMT group showed a significantly greater sIgA level than the PE group. ^∗^*P* < 0.05, ^∗∗^*P* < 0.01.

**FIGURE 5:**
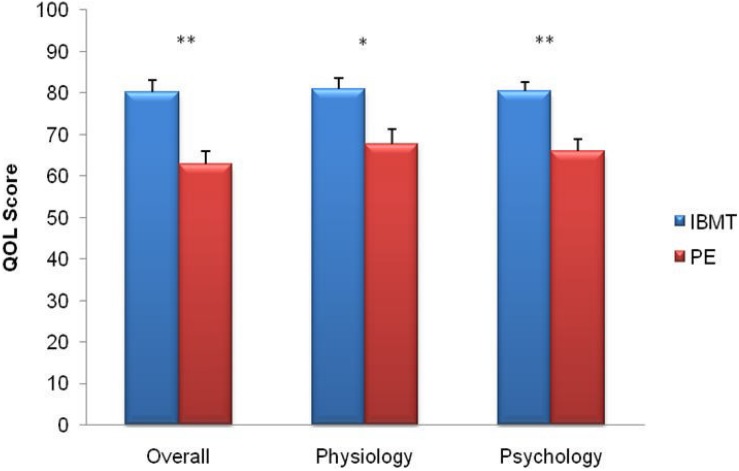
Comparison of quality of life between IBMT and PE. The horizontal axis indicates Overall Score; Physiology; Psychology. The vertical axis indicates the quality of life (QOL) score. Error bars represent standard errors. ^∗^*P* < 0.05; ^∗∗^*P* < 0.01.

The IBMT and PE groups did not differ significantly in age (*t*_53_ = 0.08, *p* = 0.94) and education (*t*_53_ = 1.11, *p* = 0.27). Following training, we assessed behavior, resting state brain activity and structural changes using fMRI and various physiological measures including HRV, SCR, sIgA and cortisol. Significant differences between the two groups are discussed in this section and in Figures 1–5.

### Brain Imaging

Based on previous studies of long-term meditation effects, we chose dorsal ACC (dACC) as the seed region and performed a whole brain functional connectivity analysis using resting state fMRI (Raichle et al., 2001; Fox et al., 2005). A significant correlation between dACC and striatum was detected in the combined IBMT and PE groups (*N* = 26, 13 in each group). Crucially, within the striatum, there was a significant difference in functional connectivity between the two groups (*P*_FWE_ < 0.05, small volume corrected); with a greater functional connectivity in the IBMT group see Figures 1A,B. When the statistical threshold was lowered (to an uncorrected level of *P* < 0.001), stronger functional connectivity between the dACC and insula was found in the IBMT group, relative to the PE group. In terms of structural differences, we found increased gray matter volume in striatum (left caudate and right putamen) using VBM in the IBMT group compared to the PE group (*P*_FWE_ < 0.05, corrected), see Figure 2. No other significant differences were found at this significance level. However, when the statistical threshold for VBM analysis was lowered to a descriptive level (uncorrected *p* < 0.005), PE showed increased gray matter in parietal and sensory-motor cortex relative to IBMT.

### Physiology

We divided the data into four periods in Figures 3A,B: 5-min baseline with eyes open (labeled as baseline 1), two 10-min periods of rest with eyes closed (labeled as period 2 and 3), and 5-min post baseline with eyes open (labeled as baseline 2), and recorded physiological indexes including heart rate, SCR, abdomen and chest respiratory amplitude for each subject in the two groups. The ANOVAs showed lower resting (baseline) SCR (*F*_1_,_38_ = 2.48, *p* = 0.123), significantly lower SCR following the two 10-min resting (periods 1 and 2) and post baseline periods separately (*F*_1_,_38_ = 8.42, *p* = 0.006; *F*_1_,_38_ = 5.25, *p* = 0.028; *F*_1_,_38_ = 5.58, *p* = 0.023).

High-frequency HRV was greater in the IBMT group than in the PE group following the second 10-min resting period (period 2) and during post baseline (*F*_1_,_33_ = 4.41, *p* = 0.043; *F*_1_,_33_ = 8.02, *p* = 0.008), The IBMT showed better autonomic regulation of SCR and high-frequency HRV, see Figures 3A,B. Results of lower SCR and more high-frequency HRV demonstrated better ANS regulation, especially greater parasympathetic activity after 10 years of IBMT.

The PE group showed significantly lower resting heart rate (*F*_1_,_38_ = 8.48, *p* = 0.006) and greater chest respiratory amplitude than the IBMT group (*F*_1_,_34_ = 5.41, *p* = 0.026), indicating the training effects on cardiovascular system, see Figure 3C.

### Immune Function

Repeated-measures ANOVAs were conducted with the factor of Group (IBMT and PE), and the within-subjects factor of Session (labeled as Before Stress, After Stress, and Additional 20-minute in Figure 4). The analyses revealed significant main effects for Group (*F*_1_, _53_ = 14.229, *p* < 0.01) and Session (*F*_2_, _106_ = 32.31, *p* < 0.001), as well as a significant interaction for Group × Session (*F*_2_, _106_ = 8.636, *p* < 0.01). The long-term IBMT group showed higher basal sIgA level (*p* < 0.05) compared to PE before stress. After stress, sIgA in both groups increased, but IBMT was significantly higher than PE (*F*_1_,_53_ = 9.628, *p* < 0.01). After the additional 20-min practice, the sIgA in IBMT group continued to increase whereas it decreased in PE group, producing a significantly higher sIgA 20 minutes after stress (*F*_1_,_53_ = 20.779, *p* < 0.001). The IBMT group also showed marginally less basal cortisol concentration prior to stress than did PE (*p* = 0.07).

### Quality of Life

Independent sample *t*-test showed significantly higher ratings in physical and psychological domains and in overall score of quality of life in the IBMT group compared to the PE group (*t*_53_ = 3.02, *p* = 0.004; *t*_53_ = 4.17, *p* = 0.000 and *t*_53_ = 4.27, *p* = 0.000); see Figure 5.

## Discussion

We observed similar default mode brain networks in both groups at resting state, as reported by previous studies (Raichle et al., 2001). Consistent with our first hypothesis – and with previous results summarized in the introduction – the IBMT group showed stronger dACC-Striatum functional connectivity at rest. This finding may relate to differences in parasympathetic regulation (Tang et al., 2009, 2019). In support of this view, the groups showed different physiological indexes of HRV and SCR indicative of parasympathetic reactivity and immune function, with the IBMT group showing generally greater HF-HRV and sIgA levels, and lower SCR (Tang et al., 2009, 2010). In addition, self-ratings in the quality of life scale were superior for the IBMT group.

In contrast, the PE group showed lower resting heart rate and greater chest respiratory amplitude compared to the IBMT group. These findings for the exercise group are in line with previous findings of exercise improving the cardiovascular system and health (Okazaki et al., 2005; Hillman et al., 2008). They also suggest different brain and physiological biomarkers for PE and IBMT, reflecting somewhat different underlying mechanisms in long-term physical exercise in comparison with meditation. This may indicate the potential for integration of the two forms of practice.

### Central Nervous System

Strong self-discipline and regulation are required to maintain 10 years of practice. In our previous study (Tang et al., 2009), we found increase in ventral midfrontal brain system control over parasympathetic activity after 5 days of IBMT. This beginning stage of practice requires ventral ACC coordination with the parasympathetic nervous system to change the participant’s state. Members of the IBMT group also reported a happy and enjoyable experience during and after training. These reports may further suggest involvement of the striatal reward system (Tang, 2017).

Dopamine pathways serve the functions of reward (motivation), pleasure, and euphoria. The putamen and caudate play a key role in reinforcement and implicit learning and are thus associated with reward (Packard and Knowlton, 2002; Dahlin et al., 2008). Longitudinal shrinkage of the whole striatum with age is evident, even in a selected group of healthy adults (Raz et al., 2003). The IBMT group seemed to reverse this aging effect by showing larger putamen and caudate volume than the PE group, consistent with previous findings of practice-related neuroplasticity and improved connectivity of the striatum with the ACC (Draganski et al., 2004; Mahncke et al., 2006; Tang et al., 2009). These effects may be of practical importance in improving quality of life and brain health during aging.

With regular IBMT practice, the participants achieved greater satisfaction (as reported in their ratings), and this positive mood and reward experience may motivate the participants to maintain practice. It was reported that emotion and motivation associated with reward experiences serve to direct executive control and enhance overall behavioral performance (Pessoa, 2009). This also could help explain the persistence of long-term IBMT practice. For the PE participants, their motivator would be the desire for physical fitness, which may particularly motivate their long-term practice. It should also be noted that Chinese collectivist culture may also facilitate group practice in local communities where old adults easily got together for shared daily activities (Goodwin, 1999; Nisbett, 2004; Markus and Conner, 2014).

When the statistical threshold was lowered (to an uncorrected level of *p* < 0.001), stronger dACC and insula functional connectivity was found in the IBMT relative to the PE group. The dACC and insula are thought to work together in resting state (Dosenbach et al., 2007) and both have been shown to be involved in high-level attention and self-regulation (Posner et al., 2007; Sridharan et al., 2008). Moreover, the ACC and insula have been linked to self-regulation in connectivity studies (Posner et al., 2007; Sridharan et al., 2008). Our finding of increased functional connectivity between the dACC and insula is consistent with the distribution of Von Economo neurons in these two brain areas and their connectivity in the resting state (Allman et al., 2001; Dosenbach et al., 2007; Tang et al., 2015b). We propose that these two regions may provide an anatomical base for successful self-regulation (Tang, 2017) that may have close relationships to interoceptive inference (Seth and Friston, 2016).

Our current findings suggest that IBMT operates via central nervous system control of peripheral responses to enhance self-regulation. On the other hand, in our study PE appears to train the cardiovascular system more than IBMT. Thus, the two types of practice appear to involve distinct body-brain mechanisms. We failed to find significant gray matter changes in PE compared to IBMT (*P*_FWE_ < 0.05, corrected). However, when the statistical threshold for VBM analysis was lowered (by using an uncorrected *P* < 0.005), PE showed more gray matter in parietal and sensory-motor cortex than IBMT. The tendency for more sensory motor activity in PE might reflect more attention to the body during exercise and is also consistent with previous findings using relaxation training (Tang et al., 2009; Tang, 2017). These results are consistent with recent findings that continuous light-intensity physical activity contributes to brain plasticity (Spartano et al., 2019).

### Physiology

Physiological measures of heart rate, SCR, respiratory amplitude and rate, and HRV are biomarkers of autonomic regulation (Pumprla et al., 2002; Borresen and Lambert, 2008; Tang et al., 2009). The IBMT group showed significantly better physiological reactions in lower SCR and higher abdominal respiratory amplitude than the PE group. These results reflected greater ANS regulation in the IBMT group. The greater high-frequency HRV in the IBMT group after training indicates successful inhibition of sympathetic tone and activation of parasympathetic tone in comparison with the PE group (Tang et al., 2019). In contrast, lower resting heart rate and increased chest respiratory amplitude were found in the PE group, congruent with physical exercise training which mainly engages cardiovascular system of the body (Pumprla et al., 2002; Borresen and Lambert, 2008; Hillman et al., 2008). These results were consistent with previous findings of endurance exercise effects on autonomic control of heart rate (Okazaki et al., 2005; La Rovere and Pinna, 2014).

Five days of IBMT improves attention and self-regulation by changing central and autonomic nervous system interaction, and 1-month of continuous IBMT practice shows accumulated effects in a dose dependent fashion (Fan et al., 2010, 2013; Tang, 2017). Further, about 10 h IBMT over 1-month period increases white matter connectivity in ACR, key regions associated with ACC and other areas (Tang et al., 2010). Months to years of aerobic exercise improve physical health and cognitive performance (Hillman et al., 2008). The current 10-year training did not examine changes after varying periods of time. Thus, we do not know when and how much training is optimal; these questions will require further research.

### Immune Function

Secretory immunoglobulin A (sIgA), an index of mucosal immunity, plays an important role in host defense. The secretory immune system of the upper respiratory tract’s mucosal tissues is considered as the body’s first line of defense against pathogens. Salivary sIgA becomes a focus of interest in psychoimmunological research since it has been shown to be sensitive to variations in subjective and objective stress levels (Fan et al., 2010). Prior research had shown that an additional training session immediately after acute stress increased release of salivary sIgA in a group trained with 5-day IBMT in comparison to a control group given the same amount of relaxation training. Additionally, 4 weeks of increasing amounts of IBMT significantly increased basal sIgA level, suggesting further improvements in mucosal immune function. As we predicted, the long-term IBMT group had a greater sIgA level than the PE group. This higher immune function in an aging population may be of importance in maintaining health (Tang, 2017).

Our results and explanations must be considered in the context of several limitations. First, a relatively small sample of subjects with more females was recruited following the 10-years of training, so we could not answer the questions of sex-differences and dispositional influences (Killgore and Yurgelun-Todd, 2001; Gross, 2007; McRae et al., 2008; Tang et al., 2016). Future large trials will allow us to explore the sex-differences in behavioral, physiological and brain changes following longitudinal practice. Second, our sample was from a Chinese aging population; although our American adult results showed the same underlying mechanism for IBMT (Tang et al., 2012, 2015a; Tang, 2017). Replication of these findings would be helpful in a western aging population. Third, our design did not include no-training group. Since both PE and IBMT had already been shown to have benefits, it did not seem ethical to have a group with no activity for 10 years. However, this design may generate additional valuable information on how the IBMT and PE training postpone the normal aging process compared to a waitlist group. Moreover, future research should have assessments before and after the 10-years of training and examine differences at varying points during training since we do not know when and how much training is optimal. It should be noted that our previous research has reported mindfulness effects on attention, cognitive performance, emotional states and stress regulation. In the current study, we aimed to further explore physical and psychological changes of quality of life using widely validated WHO Quality of Life Survey. Future research should include multi-faceted questionnaires to fully evaluate the potential changes. Since our study findings are related to Alzheimer’s Disease (AD) biomarkers, future intervention studies could clarify any differential response to the interventions in older adults with and without preclinical AD–and any effects of treatment on these AD biomarkers themselves in rigorous, randomized controlled trials. Finally, future studies should examine the benefits of combining PE and IBMT to determine if combining training methods would lead to further benefits. Overall, our study represents an important extension of previous research on the effects of physical exercise and meditation practice on the aging process.

In summary, the present findings suggest that the differences between long-term mindfulness practice and physical exercise may manifest in the functional architecture of this circuit including ACC, striatum and the parasympathetic branch of the autonomic nervous system. These differences are accompanied by higher quality of life ratings and immune function in the IBMT group. In contrast, PE group showed lower resting heart rate and increased chest respiratory amplitude, congruent with the notion that physical exercise training mainly engages and trains the cardiovascular system of the body. These results were also consistent with previous findings of endurance exercise effects on autonomic control of heart rate. Since the mechanisms of PE and IBMT are partially distinct, it is feasible to integrate physical and mental training to achieve greater health and well-being.

## Data Availability Statement

The raw data supporting the conclusions of this article will be made available by the authors, without undue reservation, to any qualified researcher.

## Ethics Statement

This study was carried out in accordance with the recommendations of DUT Institutional Review Board. The protocol was approved by the DUT Institutional Review Board. All subjects gave written informed consent in accordance with the Declaration of Helsinki.

## Author Contributions

Y-YT designed the study. YF, QL, and Y-YT performed research and analyzed data. Y-YT, L-HT, RT, KF, RK, MP, BT, KC, and ER interpreted the data and wrote/edited the manuscript.

## Conflict of Interest

The authors declare that the research was conducted in the absence of any commercial or financial relationships that could be construed as a potential conflict of interest.

## Footnotes

1 http://www.fil.ion.ucl.ac.uk/spm

2 http://dbm.neuro.uni-jena.de/vbm

3 www.fil.ion.ucl.ac.uk/spm/ext
